# Characterization of the 5′-flanking region of the human DNA helicase B (*HELB*) gene and its response to *trans*-Resveratrol

**DOI:** 10.1038/srep24510

**Published:** 2016-04-15

**Authors:** Fumiaki Uchiumi, Jun Arakawa, Keiko Iwakoshi, Sayaka Ishibashi, Sei-ichi Tanuma

**Affiliations:** 1 Department of Gene Regulation, Faculty of Pharmaceutical Sciences, Tokyo University of Science, Noda-shi, Chiba-ken 278-8510, Japan; 2 Research Center for RNA Science, RIST. Tokyo University of Science, Noda-shi, Chiba-ken 278-8510, Japan; 3 Department of Biochemistry, Faculty of Pharmaceutical Sciences, Tokyo University of Science, Noda-shi, Chiba-ken 278-8510, Japan

## Abstract

Human DNA helicase B (HELB/HDHB) regulates DNA replication through association with human DNA polymerase α-primase. In the present study, an 866-base pair (bp) of the 5′-flanking region of the human *HELB* gene-containing Luciferase (Luc) reporter plasmid, pHDHB-Luc was transfected into various cell lines and Luc activity was analyzed. Deletion analyses revealed that a 121-bp containing the major transcription start site (TSS) was essential for the basal promoter activity in all tested cells. TF-SEARCH analysis indicated that GC-box/Sp1 and duplicated GGAA-motifs containing putative STAT-x and c-ETS binding sites are located close to the TSS. Furthermore, chromatin immunoprecipitation (ChIP) analysis showed that PU.1 and Sp1 bind to the 121-bp region. Reverse transcriptase-polymerase chain reaction (RT-PCR) and western blot analyses showed the *HELB* gene and protein expression was up-regulated by *trans*-Resveratrol (Rsv) treatment in HeLa S3 cells. Moreover, transfection experiment indicated that mutations on the GC-boxes and the duplicated GGAA-motif greatly reduced promoter activity and the response to Rsv in HeLa S3 cells. These results suggest that Rsv, which is a natural compound that has been found to elongate the lifespan of various organisms, regulates *HELB* promoter activity through co-operation of the GC-boxes and the duplicated GGAA-motif in the 121-bp.

DNA helicases are DNA-binding proteins that have various functions such as unwinding DNA double-strands or changing chromatin structures. Thus, they regulate DNA replication, repair, recombination and transcription, and these nuclear functions in turn affect cellular proliferation, differentiation and senescence^1^,^2^.

Analysis of the amino acid sequence and cellular functions of mouse DNA helicase B (Helb) revealed that it plays important roles in regulating mammalian cells^3^. A temperature-sensitive mutant defective in DNA replication from the mouse mammary carcinoma cell line showed diminished major DNA-dependent ATPase activity during incubation at the non-permissive temperature^4^. This resulted from a mutation in the mouse *Helb* gene encoding a DNA homologous to a bacterial Rec D^5^. The human *HELB* (*HDHB*) gene encodes a DNA-replication-associated helicase^6^. The dominant negative mutant of the human HELB, which lacks ATPase and helicase activity, inhibited DNA synthesis when it was micro-injected into the nucleus of cells at the early G_1_ phase^6^. Moreover, it has been shown that HELB localizes in nuclear foci induced by DNA damaging agents and that the localization is regulated by CDK phosphorylation^7^. Although, the *Helb* gene is highly expressed in the testis and thymus^6^, the mechanism by which the expression of the *Helb*/*HELB* gene is regulated has not been fully understood.

Recent studies have suggested that cellular senescence is regulated by DNA-repair systems, including the maintenance of telomeres^8^,^9^. For example, it has been shown that mutations on the *WRN* gene, which encodes a Rec Q helicase, cause Werner’s syndrome^10^. We previously observed that human *WRN* gene expression (and its promoter activity) is induced in HeLa S3 cells by treatment with *trans*-Resveratrol (Rsv)^11^, which is a natural compound that has been found to have the beneficial effect of elongating lifespan in various organisms^12^,^13^. We also observed that Rsv up-regulated expression of the *HELB* and *TP53* gene^11^,^14^. Given that the p53 mainly acts as a “guardian of the genome”, the *HELB* gene might be synchronously regulated by Rsv to control DNA-repair system appropriately. However, at present, it has not been shown how Rsv affects transcription of DNA repair factor-encoding genes. Therefore, it is worth to assess the molecular mechanism of how the *HELB* gene responds to the natural compound Rsv.

In the present study, we isolated a promoter-functional 866-base pair (bp) fragment of the 5′-flanking region of the human *HELB* gene by PCR using genomic DNA from the HL-60 cells as a template. Sequence analyses and primer extension experiments revealed that the *HELB* promoter lacks a TATA box but possesses a single major transcription start site (TSS). Deletion and mutation analyses suggested that GC-box/Sp1 elements and a duplicated GGAA motif, containing overlapping putative STAT-x and c-ETS binding sites, are essentially required for promoter activity with positive response to Rsv in HeLa S3 cells. Chromatin immunoprecipitation (ChIP) analyses indicated that Sp1 and PU.1 bind to the 121-bp containing the major TSS. Increases in the *HELB* gene transcript levels and its encoding protein levels after Rsv treatment were confirmed by reverse transcriptase-polymerase chain reaction (RT-PCR) and western blot analyses, respectively. Taken together, these data suggest that the GC-boxes and the duplicated GGAA motif in the 121-bp region play important roles in the regulation of *HELB* promoter activity in response to Rsv.

## Results

### Isolation of the 5′-flanking region of the human *HELB* gene

**Figure 1:**
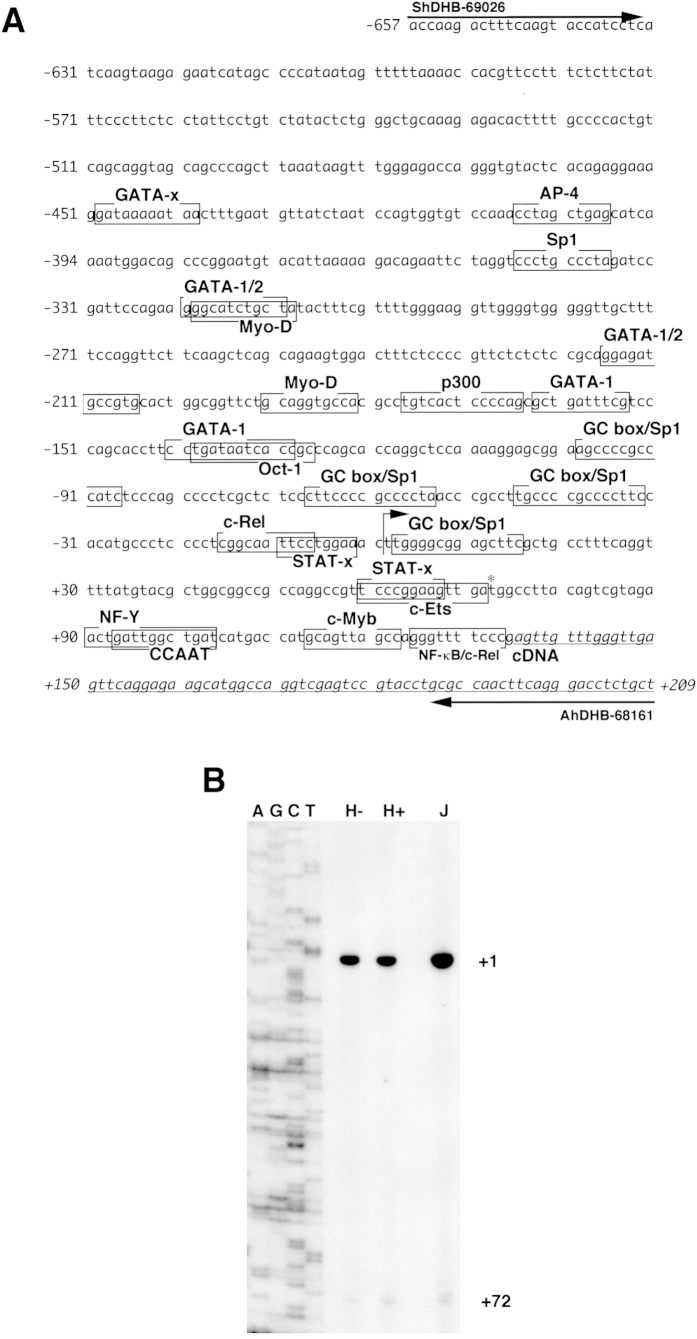
Characterization of the 5′-flanking region of the human *HELB* gene. (**A**) Nucleotide sequence of the 866-bp 5′-flanking region of the human *HELB* gene, which was obtained from PCR, is shown. The major transcription start site determined by primer extension analysis (Fig. 1B) is designated nucleotide +1 of the gene (large arrow). The minor transcription start site is indicated with an asterisk. Putative transcription factor-binding sites (TFSEARCH score > 85) are shown as boxes. (**B**) Primer extension analysis of the human *HELB* transcripts. Total RNAs (10 μg) from HL-60 cells treated with or without 12-*O*-tetradecanoyl-phorbol-13-acetate (TPA) (lanes H+ and H−, respectively), and Jurkat (lane J) cells (1 × 10^7^) were hybridized to a ^32^P-labeled AhDHB-68161 primer (1 × 10^6^ cpm) in 1 × hybridization buffer (15 μL) at 65 °C for 90 min, 42 °C for 3 min, 37 °C for 3 min, and 25 °C for 3 min. Ethanol-precipitated nucleotides were then treated with RTase at 42 °C for 1 h. RNase-treated DNAs were analyzed on 8 M urea-6% polyacrylamide gels. Sizes of extended primers were determined by comparison with the adjacent genomic sequence, which was generated by the same primer (^32^P-labeled AhDHB-68161) with pGL4-HDHB as a template (lanes AGCT). The gel was exposed to X-ray film for 7 days (lanes H−, H+, and J) or 12 h (lanes AGCT).

The nucleotide sequence of the 866-bp region (Fig. 1A) contained in the pGL4-HELB construct is identical to that of Sequence ID: ref|NW 004078075.1| (nucleotide no. from 28831901 to 28832766) and ref|NT 029419.12| (nucleotide no. from 28838871 to 28839736), which possesses the 5′ end of the human *HELB* cDNA (GENE ID, 92797 HELB on chromosome 12).

To identify the TSS for the *HELB* mRNA, primer extension analysis was performed (Fig. 1B). The primer AhDHB-68161was 5′-end labeled with ^32^P and hybridized to total RNA isolated from HL-60, and Jurkat cells. Major and minor DNA fragments were extended from the labeled primer (Fig. 1B). The major TSS is designated nucleotide number +1. That of the minor band is +72 in the *HELB* promoter region.

The TF-SEARCH program (http://www.cbrc.jp/research/db/TFSEARCH.html) was used to search the 866-bp region for characteristic recognition sequences of several known transcription factors (Fig. 1A). Putative transcription factor-binding elements GATA-1, GATA-2, GATA-3, AP-4, MYO-D, p300, OCT-1, GC-box/Sp1, c-REL/NF-κB, STAT-x, NF-Y, CCAAT, c-MYB and c-ETS are suggested to be contained in the 450-bp region upstream of the TSS (Fig. 1A). The duplicated GGAA motif (5′-GGCCG**TTCC**C**GGAA**GTTGA-3′), which is located downstream of the major TSS, contains overlapping putative STAT-x and c-ETS binding elements. Interestingly, the duplicated GGAA motif is also located very close to the TSS (5′-GGCAA**TTCC**T**GGAA**ACTTG-3′). Twelve of these two 19-bp (63%) are identical, suggesting that they might make a cruciform-like structure.

### Characterization of the 5′-flanking region of the human *HELB* gene

**Figure 2:**
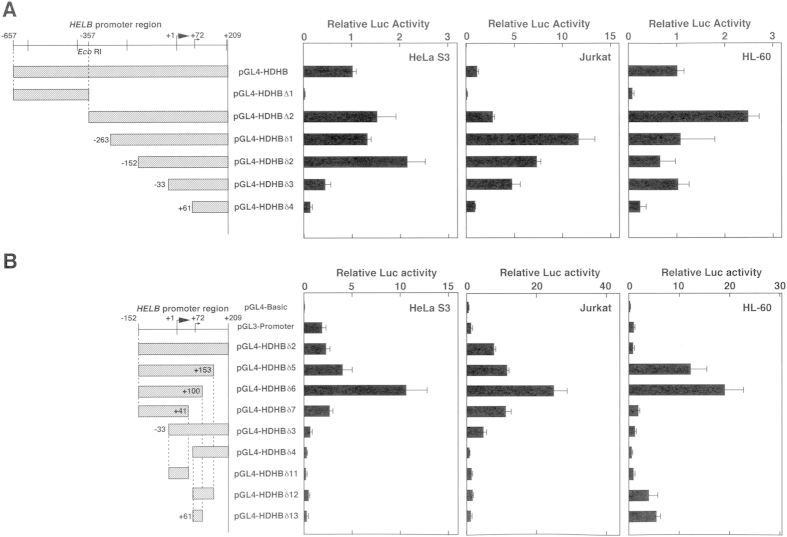
Deletion analysis of the 5′-flanking region of the human *HELB* gene. (**A**) Reporter Luc plasmids (1 μg each) pGL4-HDHB, pGL4-HDHB∆1, pGL4-HDHB∆2, pGL4- HDHBδ1, pGL4-HDHBδ2, pGL4- HDHBδ3, and pGL4- HDHBδ4 were transfected into HeLa S3, Jurkat, and HL-60 cells. Cells were collected 24 h after transfection, and Luc assays were performed. Histograms represent Luc activities relative to those of pGL4-HDHB-transfected cells. (**B**) Deletion constructs (illustrated on the left) were transfected into HeLa S3, Jurkat, and HL-60 cells, and experiments similar to those described above were performed. Results show means ± S.D. from three independent assays.

To examine the promoter activity of the 866-bp 5′-flanking sequence of the *HELB* gene, pGL4-HDHB was transfected into HeLa S3, Jurkat, and HL-60 cells (Fig. 2A). The Luciferase (Luc) activity of pGL4-HDHB-transfected HeLa S3, Jurkat, and HL-60 cells was 0.8, 5, and 0.3-fold that of the pGL3-promoter vector-transfected cells, respectively. The results suggested that the isolated 866-bp fragment contains a functional promoter region.

To identify DNA sequences that are essential for the *HELB* promoter, several deletion constructs were introduced into HeLa S3, Jurkat, and HL-60 cells and a Luc assay was performed (Fig. 2A). Promoter activity was detected with a deletion from 5′-upsteam to nucleotide position −152 (pGL4-HDHBδ2) and −33 (pGL4-HDHBδ3) in Jurkat cells. In HeLa S3 cells, no significant Luc activity was observed in pGL4-HDHBδ3-transfected cells. Thus, the sequence between −152 and +209, which contains the GC-box/Sp1 binding sites, duplicated GGAA-motifs, and the major TSS, is primarily required for the *HELB* promoter activity.

To determine the minimal promoter sequence between −152 and +209, we made additional deletion constructs, which were transiently transfected into HeLa S3, Jurkat, and HL-60 cells (Fig. 2B). Promoter activity in HeLa S3 and HL-60 cells was greatly reduced with a deletion from +42 to +100 (compare Luc activity of pGL4- HDHBδ6-transfected cells with pGL4- HDHBδ7-transfected cells), suggesting that the 58-nt region contains essential sequences for transcription in these cell lines. However, Luc activity was detectable enough from the pGL4-HDHBδ7 transfected Jurkat cells, indicating that the region from +42 to +100 is not essential in Jurkat cells. Given that Luc activity was not observed in pGL4- HDHBδ11-transfected cells, it can be assumed that the 74-bp region from −33 to +41 is insufficient for promoter function. These results suggest that the minimal core promoter of the human *HELB* gene is contained in the 252-bp region from −152 to +100.

**Figure 3:**
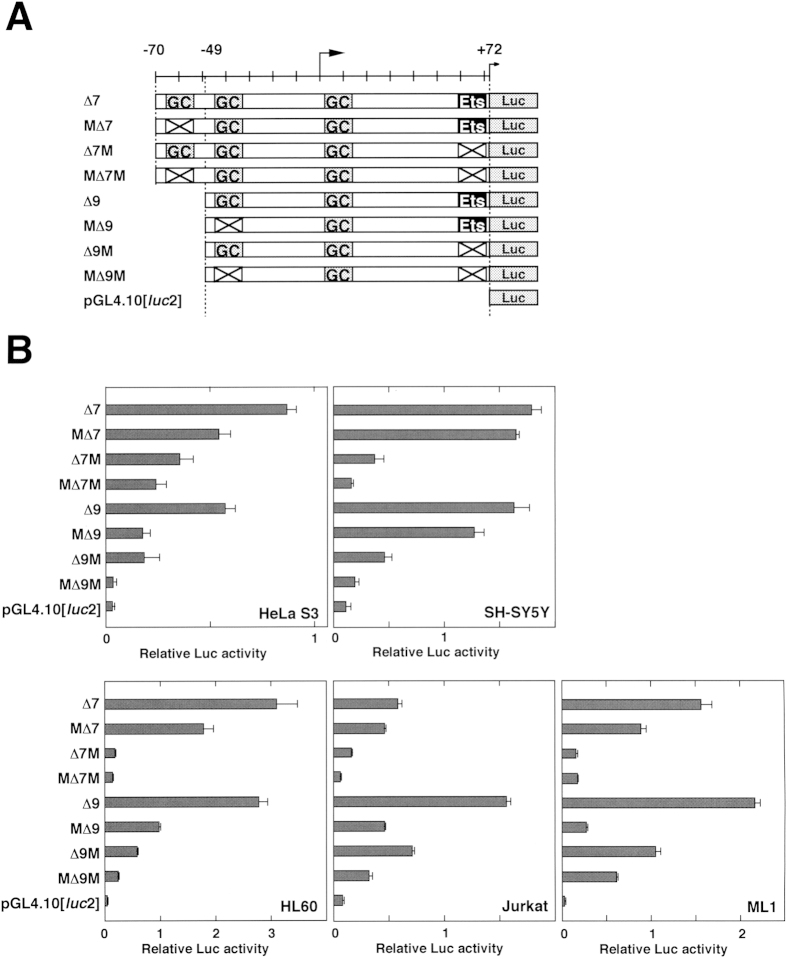
Mutation analysis of the GC-box/Sp1-binding elements and ETS family-binding motifs in the *HELB* promoter. (**A**) Schematic illustration of Luc expression plasmids carrying wild-type and mutated *HELB* promoter. Putative GC-box/Sp1-binding elements and c-ETS element in the 142-bp region are indicated. Mutated elements are shown by X. (**B**) Luc reporter plasmids (1 μg) were transfected into HeLa S3, SH-SY5Y, HL-60, Jurkat, and ML1 cells. After 24 h of transfection, cells were collected and Luc assay was performed. Horizontal bars show relative Luc activities in comparison to pGL4-HDHB vector transfected cells. Three independent experiments were performed. Results show means ± S.D.

To narrow the minimal core promoter region to the essential elements, we made further deletions and mutations (Fig. 3A). Luc activities from pGL4-∆7 and pGL4-∆9-transfected cells showed apparent value in all of the utilized cell lines (Fig. 3B), indicating that the121-bp from −49 to +72 comprises a functional promoter. This 142-bp region contains three GC-boxes (from −68 to −55, −46 to −33, and +2 to +15) and the duplicated GGAA-motif with overlapping STAT-x and c-ETS element between +59 and +71. The introduction of a mutation in one of the GC-boxes (from −68 to −55) in M∆7 caused a slight, but not severe, decrease in promoter activity in comparison to the ∆7 construct (Fig. 3B). Luc activities, however, were almost abolished by the mutation on the c-ETS element in all of the tested cells (compare ∆7 with ∆7M or M∆7M, Fig. 3B), suggesting that the *HELB* promoter activity is mainly regulated by the duplicated GGAA element between +59 and +71. Next, mutations were introduced in the second GC-box (from −46 to −33) and the duplicated GGAA-motif in the ∆9 construct (M∆9, ∆9M, and M∆9M). Surprisingly, the effect of the mutation in the GC-box was more prominent than that observed in the c-ETS element in HeLa S3, Jurkat, and ML1 cells (Fig. 3B). Introduction of double mutation (M∆9M) caused very low promoter activity in HeLa S3, SH-SY5Y, HL-60, and Jurkat cells. In contrast, promoter activity was apparent in the M∆9M-transfected ML1 cells. The results suggest that the duplicated GGAA-motif downstream of the TSS is under the control of the GC-box (from −68 to −55) and that the effect is dependent on the cell line.

### PU.1 and Sp1 form a complex with the 121-bp minimal *HELB* promoter in HL-60 cells

**Figure 4:**
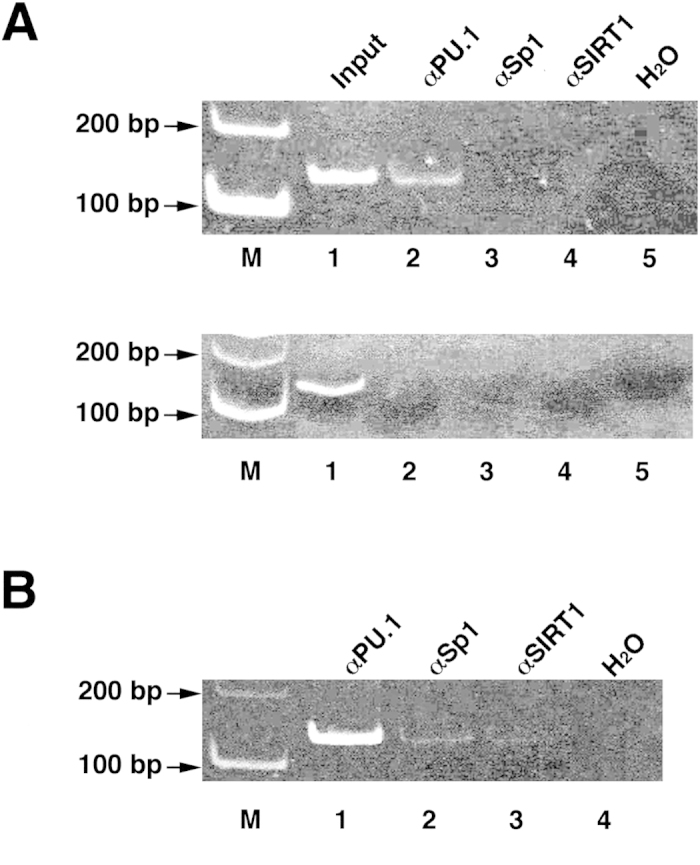
Binding of PU.1 to the GGAA containing element of the human *HELB* promoter. (**A**) HL-60 cells were collected and isolated nuclei were subjected to EZ-Zyme (Millipore Upstate) treatment. Immunoprecipitation was then performed with anti-PU.1 (lane 2), anti-Sp1 (lane 3), or anti-SIRT1 (lane 4) antibodies. PCR was performed with SHDHB-68418 and AHDHB-68297 (upper panel) or hS-1750 and AhS-1611 (lower panel) primer sets (32 cycles). Input fraction was used as a template for PCR indicating the correct size of the amplified DNA (lane 1). Lane 5 represents PCR without template double-stranded DNA. Lane M represents 100- and 200-bp fragments of the DNA ladder marker. (**B**) A similar experiment to that described above was performed with 33 cycles to amplify the 121-bp region of the *HELB* promoter.

From the results of transient transfection experiments, we tentatively determined the minimal *HELB* promoter as the 121-bp from −49 to +72, containing two GC-boxes and the c-ETS element, which consists of duplicated GGAA motifs (Fig. 1A). Thus, we speculated that either a GC-box or duplicated GGAA -motif binding proteins associates with the core *HELB* promoter region. It has been known that the GC-box is recognized by Sp1^15^. In addition, we have demonstrated that PU.1 binds to the minimal promoter regions of the human *PARG*^16^ and *IGHMBP2*^17^ genes, which contain duplicated GGAA motifs. We therefore examined the possibility that both Sp1 and PU.1 might bind to the 121-bp core promoter of the *HELB* gene by ChIP analysis (Fig. 4A). Chromatin fractions of HL-60 cells were incubated with anti-PU.1 or anti-Sp1 antibodies to show that the immunoprecipitants contain the 121-bp region (Fig. 4A, lane 2, and Fig. 4B, lanes 1 and 2). The signal from the anti-Sp1 immunoprecipitated DNA fragment was much lower than that of anti-PU.1, suggesting that the amount of the Sp1, or its binding affinity to the 121-bp, is much lower than that of the PU.1. Next, PCR was performed with primer sets to amplify nucleotides from −1750 to −1611 of the human *IGHMBP2* gene, which has no GGAA motifs. The 140-bp region was not amplified in the same manner (Fig. 4A, lanes 2 and 3). Specific DNA amplification was not detected or indistinguishable from background with the anti-SIRT1 antibody immunoprecipitated fraction used as a template (Fig. 4A, lane 4 and Fig. 4B, lane 3). These results suggest that the PU.1 and Sp1 bind to the 121-bp region but the magnitude of the association is greatly different from each other.

### Rsv upregulates expression of the *HELB* gene in HeLa S3 cells

**Figure 5:**
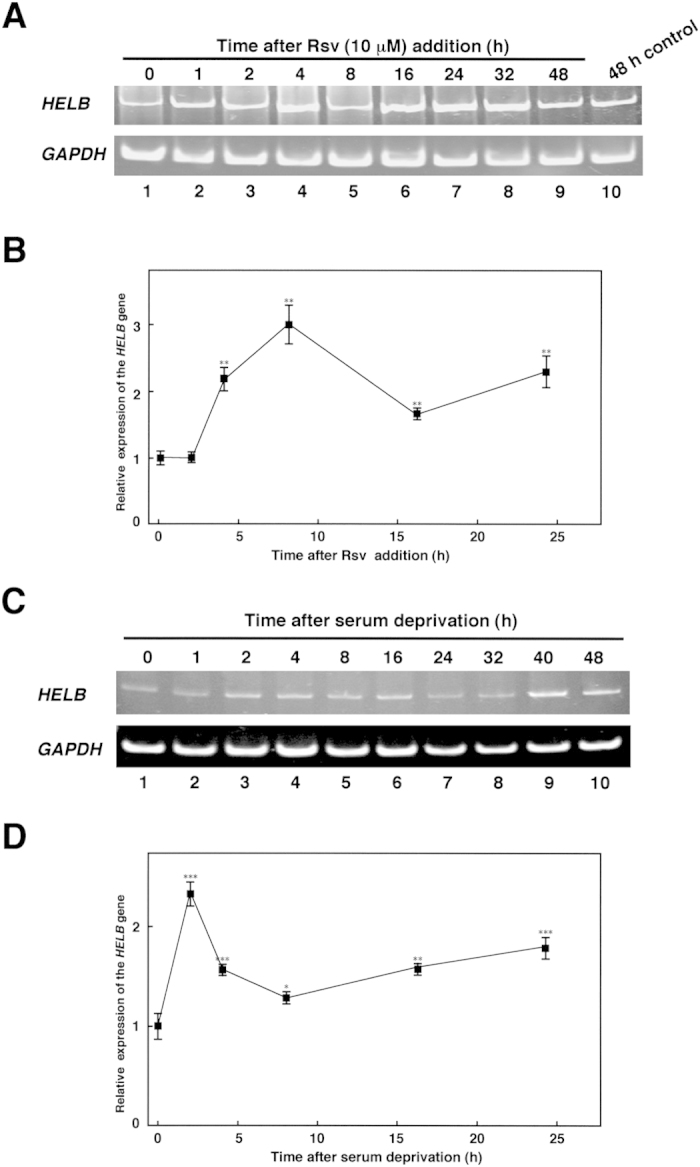
Analysis of the *HELB* gene expression by RT-PCR. (**A**) HeLa S3 cells (1 × 10^6^) were harvested after 0 to 48 h (lanes 1 to 9) of the treatment with Rsv (10 μM). Total RNAs were extracted from cells and used as templates for the reverse transcriptase reaction with a random primer. PCR products were electrophoresed on a 5% polyacrylamide gel and stained with ethidium bromide. Lane 10 represents PCR products from transcripts of untreated HeLa S3 cells. (**B**) Quantitative RT-PCR analysis was performed with total RNAs that were obtained from Rsv (20 μM)-treated HeLa S3 cells. The gene expression ratio *HELB*/*GAPDH* was calculated and results show relative values compared with that from non-treated cells. Results show means ± S.D. from three independent experiments. (**C**) Culture medium for HeLa S3 cells (1 × 10^6^) was changed to DMEM without serum. A similar experiment to that described in (**A**) was performed with primer sets to amplify *HELB* and *GAPDH* transcripts. (**D**) Similar experiment as described in (**B**) was carried out with total RNAs that were obtained from serum deprived cells. Results show relative gene expression ratio *HELB*/*GAPDH* compared with that from cells cultured with serum. Results show means ± S.D. from three independent experiments. Significance of differences between time 0 control and (**B**) Rsv treated- or (**D**) serum-deprived-cells were analyzed by Student’s *t*-test (*p < 0.05, **p < 0.01, and ***p < 0.001).

Previously, we observed that the *HELB* gene expression was induced after 24 h treatment of Rsv (10 μM) in HeLa S3 cells^11^. As expected, the time-dependent inductions of *HELB* gene expression were shown by RT-PCR analysis (Fig. 5A). The relative amount of the *HELB/GAPDH* transcripts gradually increased until 24 to 32 h after Rsv treatment (Fig. 5A, lanes 7 and 8). Quantitative RT-PCR analysis showed that the *HELB* gene expression reached a peak after 8 h of Rsv (20 μM) treatment (Fig. 5B), then it gradually declined for the next 16 h. Next, we examined whether the *HELB* transcripts are augmented by serum starvation to attenuate cell growth. The *HELB* gene transcripts increased slightly after 2 to 16 h of serum withdrawal, decreased for the next 24 h, then increased again (Figs 5C,D), suggesting that the gene expression mechanism of serum starvation differs to that of Rsv treatment. The results also showed that the expression profile of the *HELB* gene after Rsv or serum-deprivation treatment resembles to that of the *TP53* gene^14^.

### Accumulation and degradation of HELB protein in HeLa S3 Cells after Rsv treatment

**Figure 6:**
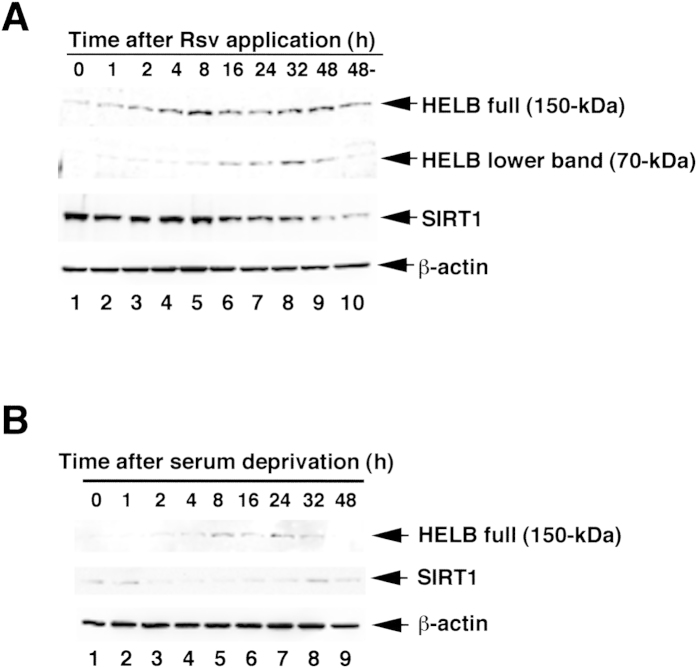
Western Blot Analysis of the HELB protein in HeLa S3 cells. (**A**) HeLa S3 cells (1 × 10^6^) were treated with 10 μM of Rsv and harvested at indicated times. Lane 10 represents a sample from cells that were harvested after 48 h without Rsv treatment. Protein extracts were separated by a SDS-PAGE. Western blotting was then performed with antibodies against HELB, SIRT1 and β-actin. (**B**) Culture medium for HeLa S3 cells (1 × 10^6^) was changed to non-serum containing DMEM and harvested at indicated times. Western blot analysis was then performed with first antibodies against HELB, SIRT1 and β-actin.

The function of the HELB protein is thought to control normal progression of cell cycle from G1 to S phase^6^. Notably, the amount of the p53 protein, which regulates G1/S transition, increases after Rsv treatment or serum starvation^14^. Hence, we performed western blotting to examine whether Rsv or serum deprivation also upregulates HELB protein level. As shown in Fig. 6A, the relative amount of full-length HELB (150-kDa) protein gradually increased and reached a peak at 8 h after Rsv (10 μM) treatment. The protein amount plateaued 16 h after the addition of Rsv to the cell culture. This HELB protein level profile might be caused by downregulation or degradation at the post-translational level, because the lower band (70-kDa), which was detected with the same antibody, increased from 1 to 48 h after the treatment. We previously observed that the *SIRT1* promoter is activated by Rsv (10 μM) treatment in HeLa S3 cells^18^. However, SIRT1 protein level did not increase but gradually declined after Rsv treatment (Fig. 6A). The SIRT1 level was transiently reduced and then gradually augmented to return to the control level at 32 to 48 h after serum deprivation (Fig. 6B, middle panel). HELB (150-kDa) protein, on the other hand, was augmented after 8 to 24 h serum deprivation, and declined further after 24 h incubation (Fig. 6B, upper panel). The results suggest that the amounts of HELB and SIRT1 proteins in HeLa S3 cells are differently controlled upon Rsv treatment or serum starvation.

### Investigation of a Rsv-responsive element in the *HELB* promoter

**Figure 7:**
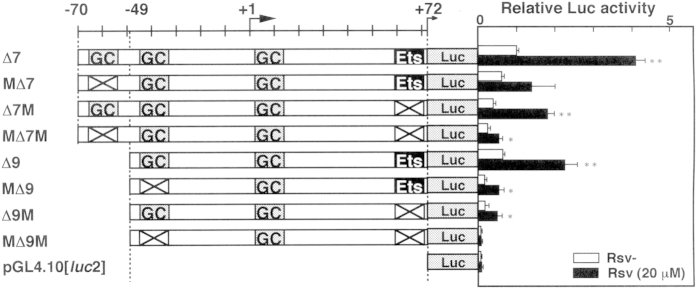
Analysis of *HELB* promoter activity after Rsv treatment. HeLa S3 cells were transiently transfected with the Luc reporter plasmids that are schematically shown on the left. After 24 h of incubation, cell culture media were changed to DMEM containing 10% FCS with Rsv (20 μM). Luc samples were prepared after further 24 h incubation. Results show relative Luc activities from these Luc reporter plasmids-transfected cells compared with the pGL4-PIF1-transfected cells. Results show means ± S.D. from three independent assays. Significance of differences between control and Rsv treated cells were analyzed by Student’s *t*-test (*p < 0.05, **p < 0.01).

In order to examine whether the GC-box/Sp1-binding motifs and c-ETS-binding element in the *HELB* promoter respond to Rsv treatment, transfection and Luc assays were performed with Luc expression plasmids carrying mutations on these elements (Fig. 3). Luc activities from pGL4-∆7 and pGL4-∆9-transfected cells were upregulated over 5-fold by the Rsv treatment (Fig. 7). The magnitude of the Rsv-induced activation of the promoter was considerably diminished by a mutation of a GC-box that is contained in the construct pGL4-M∆7, suggesting that the GC-box (−68 to −55) plays a part in the activation of the *HELB* promoter in response to Rsv. Given that the Luc activities and the response of pGL4-∆9-transfected cells to Rsv treatment are almost equal to those of the ∆7-transfected cells, the region from −70 to −50 may act as a modulator of the function of the duplicated GGAA element only when cells are treated with Rsv. The introduction of the mutation on the duplicated GGAA-motif greatly reduced the promoter activity, except in the case of the pGL4-∆7M construct. In comparing the results from transfection experiments with pGL4-∆7, −∆7M, and −M∆7M, with those of −∆9, −∆9M and −M∆9M, it was suggested that the duplicated GGAA-motif is regulated by the two upstream GC-boxes that are located between −68 and −55, and −46 and −33. In particular, the most upstream GC-box (−68 to −55) could function as an effective modulator to respond to Rsv in the presence of the duplicated GGAA-motif. The next GC-box (−46 to −33), which is required for full promoter activity in response to Rsv, is thought to be regulated under the control of the upstream GC-box (compare results from pGL4-∆7M-, pGL4-M∆7M- and pGL4-∆9M-transfected cells). Although, Luc activities from cells that were transfected with these mutation-introduced reporter plasmids were reduced, they persistently showed response to Rsv. The observation suggests the contribution of other sequence(s) in the 121-bp region. The *HELB* promoter activity and its response to Rsv was not completely lost until both of the secondary GC-box (−46 to −33) and the c-ETS binding sequence were disrupted (Fig. 7, MΔ9M), suggesting that the cooperative *cis*-function between these two elements is primarily important. Taken together, the *HELB* promoter and its response to Rsv is cooperatively regulated by the GC-boxes, c-ETS-binding element, and modulated by other sequence(s) implying that multiple transcription factors are involved in the control of the *HELB* promoter activity in HeLa S3 cells.

## Discussion

Transcription factor Sp1 binds to the GC-boxes with the consensus sequence 5′-(G/T)GGGCGG(G/A)(G/A)(C/T)-3′ or 5′-(G/T)(G/A)GGCG(G/T)(G/A)(G/A)(C/T)-3′^14^. We have reported that GC-boxes are commonly found in the 5′-flanking regions of the human *TERT* and *WRN* genes^19^. In HeLa S3 cells, the promoter activities of both genes are augmented by treatment with 2-deoxy-D-glucose (2DG) and Rsv treatment^11^,^20^. Interestingly, GC-boxes are also found in the promoter regions of most of the genes that encode the telomere-associated proteins, namely the shelterin proteins^21^. To date, it has not been shown whether Rsv affects expression of GC-box dependent genes. Rsv activates SIRT1, which is an NAD^+^ dependent deacetylase^12^. Importantly, Rsv induces a mitochondrial complex I-dependent increase in NAD^+^ molecule^22^. Metabolites, including NAD^+^ and acetyl-CoA, are known to affect the modification of chromatin-associating proteins^23^. Given that NAD^+^/NADH ratio affects the gene expression system^24^, transcriptional activation by Rsv might be the result from an increase in the NAD^+^/NADH ratio.

Both GC-boxes and Sp1 binding elements have been shown to play a part in the positive response to PPARδ^25^, suggesting that caloric restriction (CR) or energy-depleted stress might activate the transcription of genes whose promoters possess GC-boxes. In addition, the 263-bp region of the human *eNOS* promoter, which contains binding elements for Sp1and ETS family protein ELF1, responds to Rsv^26^,^27^. Co-location of GC-boxes and c-ETS binding elements has not only been identified in the human *SIRT1* and *TERT* promoter regions^18^,^28^, but also in the 5′-flanking regions of the human *VE-cadherin* (*CDH5*)^29^ and *presenilin 1* (*PS1*) genes^30^. In addition, an interactive regulatory function of Sp1 and ETS-1 has been observed in the promoter region of the murine guanylyl cyclase/natriuretic peptide receptor-A-encoding gene, *Npr1*^31^. In HeLa S3 cells, the duplicated GGAA-motif and the GC-boxes co-operatively modulate *HELB* promoter activity and its response to Rsv (Fig. 7), suggesting that *HELB* gene expression is regulated by a similar mechanism to that which drives those GC-box and duplicated GGAA motif containing promoters.

The implications of the duplicated or overlapping GGAA motifs on biological function have been reviewed and summarized^32^. The GGAA motifs are known to be the target binding sequences for the ETS family, including at least 27 different transcription factors^27^,^33^. Duplicated GGAA motifs are frequently found within close proximity to the TSSs of the human interferon stimulated genes (ISGs)^34^, including *OAS1*, which expression is regulated by ELF-1 in response to IFN-β treatment^35^. The duplicated GGAA motifs are also frequently contained in the 5′-flanking regions of the DNA repair-associated genes^36^. It has been reported that the c-ETS elements play an important role in the regulation of human *TERT* gene expression^37^. Moreover, tandem repeated c-ETS element is present in the human *TP53* promoter region^14^,^38^. It was shown that a putative E2F-binding element co-operatively regulate the response of the *TP53* promoter to Rsv treatment^14^. The duplicated GGAA (TTCC) elements are located in the promoter regions of the human *PARP1* and *PARG* genes^16^,^39^, which encode enzymes that regulate poly(ADP-ribosyl)ation in response to DNA-damage stress^40^,^41^. Notably, p53 and these DNA repair-associated enzymes localize in mitochondria^42^,^43^,^44^.

Telomere dysfunction causes mitochondrial compromise to overproduce reactive oxygen species (ROS)^45^. The duplicated GGAA-motifs are present in the 5′-upstream regions of the *RTEL1* gene^46^, which encode telomere and DNA replication regulating helicase^47^. Replication stresses, such as UV-irradiation, camptothecin, and hydroxyurea, induce the accumulation of the human HELB (HDHB) protein on chromatin^48^, suggesting that the HELB is involved in the DNA repair or chromosomal maintenance. Although, it has not been determined whether HELB is involved in the regulation of telomeres, *HELB* gene expression might be regulated in a similar manner to that of the *RTEL1* gene. Another mammalian RecD type helicase PIF1, which localizes in mitochondria^49^,^50^, is involved in DNA-repair synthesis^51^,^52^, regulation of telomerase^53^, telomere integrity and genome stability^54^. Because no obvious duplication of the GGAA (TTCC) motifs is present within 500-bp upstream of its TSS, *PIF1* gene expression may be regulated by a different mechanism to that of the *RTEL1* and the *HELB* genes.

It has been proposed that mitochondria serve as master regulators of danger signaling to determine cell death or survival^55^. It should be noted that duplicated GGAA-motifs are frequently contained in the bidirectional promoter regions of human DNA-repair and mitochondrial function-associated genes^36^,^56^, including *TP53*, which encodes p53, the “guardian of the genome”. The Rsv upregulates *TP53* gene expression through affecting a duplicated GGAA (TTCC) motif and an E2F binding element that are present close to the TSS^14^. In this study, the CR mimetic drug Rsv was shown to induce expression of the *HELB* gene by affecting the GC-box and the duplicated GGAA-motifs in a cooperative manner. This finding suggests that the expression of the *HELB* gene is controlled in a similar fashion to the genes that encode DNA repair-, telomere maintenance- and mitochondrial function-associated factors. Recent study showed that HELB interacts with Cdc45 protein that regulates initiation of DNA replication^57^. Moreover, the HELB was shown to be involved in the control of cellular homologous recombination system^58^. These lines of evidences suggest that the HELB is a multifunctional replication/repair regulator protein. Further investigations are required to reveal the function of the HELB as a modulator of DNA repair, mitochondria, telomeres, and possibly cellular senescence.

## Materials and Methods

### Materials

The reagent Rsv was purchased from Cayman Chemicals (Ann Arbor, MI). Restriction enzymes and other DNA modifying enzymes were purchased from Takara (Kyoto, Japan) and Toyobo (Tokyo, Japan). Fetal calf serum (FCS) was purchased from Sanko Pure Chemicals, Co. (Tokyo, Japan).

### Cell cultures

HL-60 is a human promyelocytic leukemia cell line^59^. Jurkat is a human acute lymphocytic T-cell line^17^. ML-1 is a human myeloblastic leukemia cell line^60^. They were cultured in RPMI 1640 medium supplemented with heat-inactivated 10% FCS, 2 mM L-glutamine, penicillin (100 IU/mL) and streptomycin (100 μg/mL). HeLa S3 cells^20^ were cultured in Dulbecco’s Modified Eagle’s medium (DMEM), and human neuroblastoma SH-SY5Y cells^46^ were in DMEM/Ham’s F-12 medium supplemented with heat-inactivated 10% FCS, 2 mM L-glutamine, penicillin (100 IU/mL) and streptomycin (100 μg/mL).

### Construction of luciferase expression plasmids

The Luciferase (Luc) reporter plasmid pGL4-HDHB, which carries 866-bp of the 5′-flanking region of the human *HELB* promoter^20^, was further treated with restriction enzymes, blunt-ended with T4 DNA polymerase and re-ligated. The restriction enzymes (and resultant plasmids) were as follows: *Eco*RI and *Xho*I (pGL4-HDHB∆1), and *Kpn*I and *Eco*RI (pGL4-HDHB∆2).

Other deletion derivatives were generated by PCR with pGL4-HDHB as a template and various primer sets (Supplementary Table). PCR products were amplified with the following primer pairs: ShDHB-68632/AhDHB-68161, ShDHB-68521/AhDHB-68161, ShDHB-68402/AhDHB-68161, ShDHB-68309/AhDHB-68161, ShDHB-68521/ AhDHB-68217, ShDHB-68521/ AhDHB-68270, ShDHB-68521/ AhDHB-68329, ShDHB-68402/ AhDHB-68329, ShDHB-68309/ AhDHB-68217, and ShDHB-68309/ AhDHB-68270. PCR products were then digested with *Kpn*I and *Xho*I and subcloned into pGL4.10[*luc*2] vector (Promega, Madison, WI) to generate pGL4-HDHBδ1, δ2, δ3, δ4, δ5, δ6, δ7, δ11, δ12, and δ13 reporter constructs. Mutations were introduced into the GC-box/Sp1 and c-ETS binding elements, performing PCR with various sense (SHDHB-68439, SHDHB-68439M, SHDHB-68418, SHDHB-68418M) and anti-sense (AHDHB-68297 and AHDHB-68297M) primer sets (Supplementary Table). The DNA fragments of the amplified142-bp and 121-bp regions were ligated to the pGL4.10[*luc*2] vector to generate the Luc reporter plasmids pGL4-∆7, −M∆7, −∆7M, −M∆7M, −∆9, −M∆9, −∆9M, and −M∆9M.

### Primer extension analysis

Hybridization of the primer and extension reaction was performed as described previously^16^. The 30-mer nucleotide (AhDHB-68161) carrying the antisense nucleotide sequence from +209 to +186 was ^32^P-5′-end labeled with T4 polynucleotide kinase and then hybridized to total RNAs (10 μg) from HL-60, and Jurkat cells in a 1x hybridization buffer [150 mM KCl, 10 mM Tris-HCl (pH 8.3), 1 mM EDTA] at 65 °C for 90 min. After the mixture was further incubated at 42 °C for 3 min, 37 °C for 3 min, and 25 °C for 3 min, nucleotides were precipitated and then dissolved in a Reverse transcriptase buffer (25 μL) containing 0.1% DPC, 1x RT buffer, 1 mM dNTP, 50 U RNase Inhibitor (Wako Pure Chemicals Co., Tokyo, Japan), 125 U ReverTra Ace (Toyobo), and incubated at 42 °C for 60 min. Next, 100 μL of RNase reaction mixture [100 mM NaCl, 10 mM Tris-HCl (pH 7.5), 1 mM EDTA, 100 μg/mL sermon sperm DNA, 20 μg/mL RNase A] was added and the mixture was incubated at 37 °C for 15 min. The extended DNA strand from the ^32^P-labeled AhDHB-68161was ethanol precipitated and was analyzed with an 8 M urea-6% acrylamide sequencing gel.

### Transient transfection assays

Plasmid DNAs were transfected into cultured cells with the DEAE-dextran method^46^. Briefly, cells (1 × 10^6^) were treated with 0.4 mL TBS [25 mM Tris (pH 7.4), 137 mM NaCl, 5 mM KCl, 0.6 mM Na2HPO_4_, 0.7 mM CaCl2, 0.5 mM MgCl_2_] containing 2 μg of the Luc reporter plasmid and 500 μg of DEAE-dextran per mL for 30 min at room temperature. The cells were then washed with TBS to remove unadsorbed DNA and cultured for 24 h in RPMI/10% FCS. Cells were then collected and cell lysates were prepared for Luc assays, which were performed with the Luciferase assay system (Promega). The collected cells were lysed with 100 μL of 1 × Cell Culture Lysis Reagent [25 mM Tris-phosphate (pH 7.8), 2 mM DTT, 2 mM 1,2-diaminocyclohexane-N, N, N′, N′-tetraacetic acid, 10% glycerol, 1% Triton X-100], mixed, and centrifuged (12,000 × *g* for 5 sec). The supernatant was transferred to a new tube and stored at −80 °C before use in the Luc assay. Luc assay reagent (40 μL) was added to 10 μL of protein sample and mixed briefly. DEAE-dextran based multiple transfection was performed according to a protocol as described previously^46^. Chemiluminescence was immediately measured for 7.5 sec with a luminometer, Minilumat LB9506 (Berthold, Bad Wildbad, Germany). The light intensity (RLU; Relative Light Units) was referred to directly as Luc activity.

### Chip and PCR analysis

ChIP assays were performed as described previously^16^,^17^. Isolated nuclei from formaldehyde-treated HL-60 cells were further treated with EZ-Zyme (Millipore Upstate, Temecula, CA). The antibodies used for immunoprecipitation were anti-PU.1, anti-Sp1 and anti-SIRT1 (Santa Cruz Biotechnology, Santa Cruz, CA). PCR was performed with the SHDHB-68418 and AHDHB-68297primer set to amplify the 121-bp region in the *HELB* promoter, or the hS-1750 and AhS-1611 primer set to amplify part of the 5′-flanking region of the human *IGHMBP2*^17^ for a negative control reaction. The conditions for PCR to amplify 121-bp from −70 to +72 of the *HELB* promoter region, and 140-bp from −1750 to −1611 of the human *IGHMBP2* promoter^17^ were 32 to 33 cycles of 94 °C for 15 sec, 55 °C for 10 sec, and 72 °C for 15 sec.

### RT-PCR analysis

RT-PCR analysis was performed as described previously^11^,^16^,^20^. Total RNAs were extracted from HeLa S3 cells with Isogen Reagent (Wako Chemicals). cDNAs were synthesized from 5 μg of total RNA with 200 U of RiverTra Ace (Toyobo) and random primers (Takara). An aliquot of each cDNA product was used to amplify the human *HELB* and *GAPDH* transcripts by PCR. Primer pairs to amplify human *HELB* and *GAPDH* transcripts have been reported previously^11^,^20^.

Conditions for the amplification of the *HELB* and the *GAPDH* transcripts were 30 cycles of 94 °C for 15 sec, 55 °C for 20 sec, and 72 °C for 20 sec; and 19 cycles of 94 °C for 15 sec, 55 °C for 20 sec, and 72 °C for 10 sec with BIOTAQ (Bioline, London, UK), respectively. PCR products were subjected to 5% polyacrylamide gel electrophoresis, and the DNAs on the gel were stained with ethidium bromide.

### Quantitative real time PCR analysis

Real time PCR analysis was carried out using the Mx3000P Real-Time qPCR System (Stratagene, La Jolla, CA) as described previously^11^,^14^,^20^. For PCR amplification, cDNAs were amplified using Thunderbird Realtime PCR Master Mix (Toyobo) and 0.3 μM of each primer pair. The primer pairs for amplifying human *HELB* and *GAPDH* transcripts were ShHELB-2630; 5′-GCTGGCCTGGAAGTAACTGT-3′/AhHELB-2740; 5′-AACTGTTTGCTCCTCGGACC-3′ and hGAPDH556/hGAPDH642^11^,^14^, respectively. Amplification was carried out initially for 1 min at 95 °C, followed by 40 cycles (95 °C 15 sec and 62 °C 30 sec). Quantitative PCR analysis for each sample was carried out in triplicates. Relative gene expression values were obtained by normalizing C_T_ (threshold cycle) values of target genes in comparison with C_T_ values of the *GAPDH* gene using the ΔΔC_T_ method.

### Western blot analysis

Western blot analysis was performed as previously described^11^,^16^,^20^. In brief, HeLa S3 cells were homogenized with 1 × RIPA buffer and 5 μg of protein was separated by 7.5% SDS-polyacrylamide gel electrophoresis (SDS-PAGE). Proteins were blotted onto nitrocellulose filters, and first antibody reactions were performed with antibodies against human HELB and SIRT1 (Santa Cruz Biotechnology), and β-actin (Calbiochem, Darmstadt, Germany) followed by the application of horseradish peroxidase (HRP)-conjugated secondary antibody (Calbiochem). Filters were treated with West-Pico (Thermo Scientific, Rockford, IL) and signal intensities were quantified with an LAS4000 system and MultiGauge Software (Fuji Film).

## Additional Information

**How to cite this article**: Uchiumi, F. *et al.* Characterization of the 5′-flanking region of the human DNA helicase B (*HELB*) gene and its response to *trans*-Resveratrol. *Sci. Rep.* **6**, 24510; doi: 10.1038/srep24510 (2016).

## Supplementary Material

Supplementary Information
