# Supplementary material for: Characterization of the 5′-flanking region of the human DNA helicase B (HELB) gene and its response to trans-Resveratrol
Source: Sci Rep. 2016 Apr 15;6:24510. doi: 10.1038/srep24510 (PMC4832242; doi:10.1038/srep24510)
Supplement: Supplementary Information [file srep24510-s1.doc]

Characterization of the 5’-flanking region of the human DNA helicase B (*HELB*) gene and its response to *trans*-Resveratrol

Fumiaki Uchiumi, Jun Arakawa, Keiko Iwakoshi, Sayaka Ishibashi & Sei-ichi Tanuma

**Supplementary Table** Primers used for amplifying wild type and mutated 5’-upstream region of the human HELB (HDHB) gene

| Name | Sequence (from 5’ to 3’) |
| --- | --- |
| ShDHB-69026 | TCGGTACCAAGACTTTCAAGTACCATCCTC |
| ShDHB-68632 | GGGTACCTTCAAGCTCAGCAGAAGTGGAC |
| ShDHB-68521 | GGGTACCAGCACCTTCCTGATAATCACCG |
| ShDHB-68402 | GGGTACCACATGCCCTCCCCTCGGCAATTC |
| ShDHB-68309 | GGGTACCGGAAGTTGATGGCCTTACAGTCG |
| SHDHB-68439 | TCGGTACCCTTCCCCGCCCCTAACCCGCC |
| SHDHB-68439M | TCGGTACCCTTGCGCACCCCTAACCCGCC |
| SHDHB-68418 | TCGGTACCTTGCCCCGCCCCTTCCACATG |
| SHDHB-68418M | TCGGTACCTTGCGCAGCCCCTTCCACATG |
| AhDHB-68161 | ATCTCGAGCAGAGGTCCCTGAAGTTGGCGC |
| AhDHB-68217 | ATCTCGAGAACTCAACCCAAACAACTCGGG |
| AhDHB-68270 | ATCTCGAGCCAATCAGTTCTACGACTGTAAG |
| AhDHB-68329 | ATCTCGAGCGTACATAAAACCTGAAAGGC |
| AhDHB-68387 | ATCTCGAGGGGAGGGCATGTGGAAGGGGCG |
| AHDHB-68297 | ATCTCGAGATCAACTTCCGGGAACGGCCTG |
| AHDHB-68297M | ATCTCGAGATCAACTGCCGGCAACGGCCTG |
